# Accessibility to ERCP‐performing hospitals among patients with pancreatic cancer living in SEER regions

**DOI:** 10.1002/cam4.7020

**Published:** 2024-02-24

**Authors:** Anna Tavakkoli, Alaina Beauchamp, Tanushree Prasad, Hong Zhu, Amit G. Singal, B. Joseph Elmunzer, Nisa M. Kubiliun, Richard S. Kwon, Amy E. Hughes, Sandi L. Pruitt

**Affiliations:** ^1^ Division of Gastroenterology & Liver Diseases, Department of Medicine University of Texas Southwestern Medical Center Dallas Texas USA; ^2^ Harold C. Simmons Comprehensive Cancer Center, University of Texas Southwestern Medical Center Dallas Texas USA; ^3^ Peter O'Donnell Jr. School of Public Health, University of Texas Southwestern Medical Center Dallas Texas USA; ^4^ Department of Public Health Sciences University of Virginia School of Medicine Charlottesville Virginia USA; ^5^ Division of Gastroenterology and Hepatology, Department of Medicine Medical University of South Carolina Charleston South Carolina USA; ^6^ Division of Gastroenterology, Department of Internal Medicine University of Michigan Ann Arbor Michigan USA

**Keywords:** accessibility, disparities, ERCP, pancreatic cancer

## Abstract

**Background and Aims:**

The two most common interventions used to treat painless jaundice from pancreatic cancer are endoscopic retrograde cholangiopancreatography (ERCP) and percutaneous transhepatic biliary drainage (PTBD). Our study aimed to characterize the geographic distribution of ERCP‐performing hospitals among patients with pancreatic cancer in the United States and the association between geographic accessibility to ERCP‐performing hospitals and biliary interventions patients receive.

**Methods:**

This is a retrospective cohort study using the Surveillance, Epidemiology, and End Results (SEER)‐Medicare database for pancreatic cancer from 2005 to 2013. Multilevel models were used to examine the association between accessibility to ERCP hospitals within a 30‐ and 45‐min drive from the patient's residential ZIP Code and the receipt of ERCP treatment. A two‐step floating catchment area model was used to calculate the measure of accessibility based on the distribution across SEER regions.

**Results:**

7464 and 782 patients underwent ERCP and PTBD, respectively, over the study period. There were 808 hospitals in which 8246 patients diagnosed with pancreatic cancer in SEER regions from 2005 to 2013 received a procedure. Patients with high accessibility within both 30‐ and 45‐min drive to an ERCP‐performing hospital were more likely to receive an ERCP (30‐min adjusted odds ratio [aOR]: 1.53, 95% confidence interval [CI]: 1.17–2.01; 45‐min aOR: 1.31, 95% CI: 1.01–1.70). Furthermore, in the adjusted model, Black patients (vs. White) and patients with stage IV disease were less likely to receive ERCP than PTBD.

**Conclusions:**

Patients with pancreatic cancer and high accessibility to an ERCP‐performing hospital were more likely to receive ERCP. Disparities in the receipt of ERCP persisted for Black patients regardless of their access to ERCP‐performing hospitals.

## 1 INTRODUCTION

Over 64,000 cases of pancreatic cancer are estimated to be diagnosed in 2023.^1^, ^2^ With almost 50% of patients diagnosed with distant disease at the time of diagnosis in the United States, pancreatic cancer treatment often focuses on palliative interventions to prolong and improve a patient's life.^1^, ^3^, ^4^, ^5^, ^6^, ^7^

A hallmark symptom of pancreatic cancer is painless jaundice due to biliary obstruction by the mass. Endoscopic retrograde cholangiopancreatography (ERCP) and percutaneous transhepatic biliary drainage (PTBD) are the two most common interventions to treat painless jaundice from pancreatic cancer.^8^, ^9^, ^10^ Treatment of jaundice is typically required prior to starting chemotherapy without dose reduction and can also improve symptoms of pruritus and anorexia.^11^, ^12^, ^13^, ^14^ Since its first description, ERCP has become the preferred initial treatment for biliary obstruction from pancreatic cancer given decreased adverse events, greater survival, and improved quality of life.^9^, ^15^, ^16^, ^17^, ^18^

Despite known benefits of ERCP, there is emerging evidence of regional and racial variations in the receipt of ERCP versus PTBD. Risk factors associated with lower odds of ERCP receipt as the initial treatment of obstructive jaundice from pancreatic cancer include Black race and living in the Northeast.^19^ Geographic access to hospitals that perform ERCP may also be associated with receipt of ERCP versus PTBD.^20^ A number of prior studies have highlighted how geographic access to hospitals that perform pancreaticoduodenectomy varies throughout the United States and the impact that variations in pancreatic oncological care have on patient outcomes.^21^, ^22^, ^23^ However, to our knowledge, prior studies have not yet described geographic access to hospitals performing ERCP or examined whether disparities in access are associated with the receipt of ERCP treatment and clinical outcomes.

To address these gaps in the literature, we aimed to (1) characterize the geographic distribution of hospitals performing ERCP for patients with pancreatic cancer in Surveillance, Epidemiology, and End Results (SEER) regions; (2) measure access to ERCP‐performing hospitals within a 30‐min drive from a patient's ZIP Code; and (3) evaluate the association between travel accessibility of ERCP‐performing hospitals and biliary interventions for pancreatic cancer, in particular, the receipt of ERCP versus PTBD.

## 2 MATERIALS AND METHODS

### 2.1 Data sources

Our study is a retrospective cohort study using the SEER‐Medicare database for pancreatic cancer cases diagnosed from 2005 to 2013.^15^, ^24^ The SEER‐Medicare database has patient demographic and oncological data, including staging and treatments, for Medicare patients diagnosed with cancer in SEER‐defined geographic regions.^15^ The University of Texas Southwestern Institutional Review Board reviewed our study and exemption was obtained.

We used International Classification of Diseases (ICD) 9 codes, the American Common Procedure Terminology codes, and the healthcare common procedure codes to identify diagnoses and procedures in the Medical Provider Analysis and Review (MEDPAR) and outpatient files (Table S1).^15^ The MEDPAR file was used to identify hospital facilities and beneficiary residential ZIP Codes to measure geographic accessibility. The hospital file was used for hospital‐level descriptors and the census ZIP Code file was used for sociodemographic variables.

### 2.2 Study sample

We included patients with pancreatic cancer histology, based on ICD for Oncology 3 codes validated in prior publications, a pancreatic head mass, and/or evidence of biliary obstruction (Table S1).^15^, ^19^ We excluded patients with age less than 66 years, no date of diagnosis, a history of other cancer, histology other than adenocarcinoma, diagnosis at the time of death or autopsy, those patients who did not receive ERCP or PTBD, and patients who received an ERCP or PTBD before pancreatic cancer diagnosis (Figure S1).^15^ Continuous enrollment in Medicare Part A and B coverage, without concomitant enrollment in a health maintenance organization, for at least 12 months before their pancreatic cancer diagnosis and through death, or up to 12 months after their diagnosis, was required.^15^ The first procedure a patient received on or after the date of diagnosis designated them as having undergone either ‘ERCP’ or ‘PTBD’.

### 2.3 Outcomes and exposures

Our primary outcome was whether a patient received either an ERCP or PTBD after pancreatic cancer diagnosis. Our secondary outcome was overall survival after pancreatic cancer diagnosis. Our primary exposure was geographic accessibility to ERCP.

### 2.4 Primary exposure of interest: ERCP accessibility

We measured ERCP accessibility using the two‐step floating catchment area (2SFCA) method using the steps outlined below. For hospitals, the latitude–longitude locations of ERCP treatment sites were extracted from patient‐level Medicare claims data (MEDPAR and outpatient files) from 2005 to 2013 and augmented with records from the American Hospital Association and the Centers for Medicare and Medicaid Services hospital file. Hospitals were matched to ZIP Code Tabulation Area (ZCTA) using a spatial join in ArcGIS Pro version 3.0.3. For population distribution, 5‐year estimates of the number of adults in each ZCTA across the United States were downloaded from the 2009–2013 American Community Survey (ACS). A hospital‐to‐population ratio was generated using the total count of ERCP‐performing hospitals within each ZCTA divided by the adult population (ages 18 and older) drawn from the ACS 2009–2013 estimates. ZCTAs with no hospitals are represented as missing value for this measure.

We took the number of ERCP procedures performed within each unique ERCP‐performing hospital facility annually and averaged across the number of years operating during the study period. We calculated the supply‐to‐demand ratio for each hospital as a ratio of the hospital's average ERCP volume to the adult (ages 18 years and older) population within a 30‐min drive from a ZCTA centroid to the hospital location. Drive time calculations were conducted in ArcGIS Pro with ESRI's StreetMap Premium North America Street Network Database (Esri Inc. [2020]. ArcGIS Pro [Version 3.0.3]. Esri Inc. https://www.esri.com/en‐us/arcgis/products/arcgis‐pro/overview). The final measure of the accessibility to an ERCP‐performing hospital for each ZCTA was calculated using the 2SFCA methods, as described elsewhere.^25^ Next, each ZCTA was categorized into quartiles based on the distribution of 2SFCA scores for hospitals across all SEER regions combined: low accessibility, moderate accessibility, moderately high accessibility, and high accessibility. Patients residing in the highest (most accessible) quartile were defined as having high accessibility to ERCP.

We mapped the continuous accessibility measure to illustrate geographic variability in accessibility to treatment. We restricted the map to SEER registry areas because ERCP providers and patients outside of those registry areas were excluded.

### 2.5 Covariates of interest

The variables included in our study were age at diagnosis; sex; race; ethnicity; Charlson Comorbidity Index (CCI); tumor stage based on the American Joint Committee on Cancer, sixth edition; biliary obstruction, defined as having either cholangitis, pruritus, jaundice, obstructive jaundice, obstruction bile duct, or abnormal liver function after pancreatic cancer diagnosis; gastric outlet obstruction (GOO); year of the procedure; whether the patient lives in a metropolitan or non‐metropolitan area based on the 2003 Rural–Urban Continuum Code; whether the hospital was a National Cancer Institute (NCI)‐Designated Cancer Center or transplant center; the volume of ERCPs and/or PTBDs performed by the hospital during the study period (2005–2013); measure of patient accessibility within a 30‐, 45‐, and 60‐min drive of the hospital location; the percent of residents living below poverty; and the percent of persons with at least 4 years of college education.

### 2.6 Statistical analyses

We compared the distributions of continuous and categorical variables among patients who underwent either ERCP or PTBD using the Student's *t*‐test and *χ* ^2^ test. We estimated the adjusted odds ratio (aOR) of high patient access to ERCP within a 30‐, 45, and 60‐min drive to the hospital using multilevel mixed‐effects logistic regression. We estimated two multilevel models to describe the association between access to a hospital with ERCP capabilities and the receipt of the preferred ERCP treatment. Model 1 did not include CCI due to missing data. Model 2 was estimated for the subgroup of patients with available CCI data.

All estimates were adjusted for clustering of patients within hospitals and for hospitals within ZCTAs codes. STATA 15.0 (Stata Corp, College Station, TX, USA) was used for all analyses and descriptive maps were created in ArcGIS 3.0.3. Additionally, we performed a survival analysis comparing Black and White patients who underwent ERCP or PTBD within a 30‐min drive to the hospital using a Cox proportional hazard model. We censored patients at the time of death or last Medicare follow‐up on December 31, 2015. Our Cox model was adjusted for race, gender, 30‐min accessibility, stage, age at diagnosis, and comorbidities. The log‐rank test was used to compare Kaplan–Meier survival curves.

## 3 RESULTS

### 3.1 Patient characteristics

We identified 8246 patients who underwent ERCP or PTBD (Figure S1). The mean age was 77.1 years with standard deviation of 7.7. The majority of patients were female (58.0%), White (84.3%), and non‐Hispanic (92.8%). The majority (83.5%) presented with a head of pancreas mass. White patients were more likely to get ERCP while Black patients more frequently received PTBD (Table 1). While patients with GOO and stage IV disease were more likely to receive PTBD, patients treated at NCI‐Designated Cancer Centers were more likely to receive ERCP than those treated at non‐NCI cancer centers (Table 1). There was no statistically significant difference in race among patients presenting with both stage IV disease and GOO; however, Black patients were more frequently diagnosed with stage IV disease than White patients (28.8% vs. 32.0%, *p* = 0.02). Patients who received PTBD were more likely to live in ZIP Codes with >15% of residents living in poverty and in ZIP Codes with a lower proportion of college educated persons (Table 1).

**TABLE 1:** Characteristics of 8246 patients with pancreatic cancer who received ERCP or PTBD.

Variable	ERCP (*n* = 7464)	PTBD (*n* = 782)	*p*‐Value
Age at diagnosis, mean ± SD	77.1 ± 7.7	77.1 ± 8.1	0.99
Gender, *n* (%)			
Male	3135 (42.0)	329 (42.1)	0.97
Female	4329 (58.0)	453 (57.9)	
Race, *n* (%)			
White	6328 (84.8)	627 (80.2)	<0.001
Black	673 (9.0)	107 (13.7)	
Asian or Pacific Islander	419 (5.61)	41 (5.2)	
Ethnicity			
Non‐Hispanic	6936 (92.9)	717 (91.7)	0.20
Hispanic	528 (7.1)	65 (8.3)	
Charlson comorbidity Index, *n* (%)			
0	2451 (32.8)	242 (31.0)	0.72
1	1528 (20.5)	167 (21.4)	
≥2	1667 (22.2)	182 (23.3)	
Missing	1818 (24.4)	191 (24.4)	
Gastric outlet obstruction, *n* (%)			
No	7353 (98.5)	750 (95.9)	<0.0001
Yes	111 (1.5)	32 (4.1)	
Biliary obstruction, *n* (%)			
No	1312 (17.6)	175 (22.4)	0.001
Yes	6152 (82.4)	607 (77.6)	
AJCC staging, *n* (%)			
Stage 1	757 (10.1)	60 (7.7)	0.005
Stage 2	2594 (34.8)	250 (32.0)	
Stage 3	667 (8.9)	61 (7.8)	
Stage 4	2133 (28.6)	268 (34.3)	
Unknown	1313 (17.6)	143 (18.3)	
Location of pancreatic tumor			
Head of pancreas	6244 (83.7)	641 (82.0)	0.47
Body/tail pancreas	172 (2.3)	21 (2.7)	
Unknown	1048 (14.0)	120 (15.4)	
Procedure year, *n* (%)			
2005	824 (11.0)	75 (9.6)	0.014
2006	804 (10.8)	107 (13.7)	
2007	806 (10.8)	99 (12.7)	
2008	857 (11.5)	95 (12.2)	
2009	850 (11.4)	102 (13.0)	
2010	890 (11.9)	96 (12.3)	
2011	895 (12.0)	80 (10.2)	
2012	834 (11.2)	68 (8.7)	
2013	704 (9.4)	60 (7.7)	
National Cancer Institute Cancer Center, *n* (%)			
No	6501 (87.1)	705 (90.2)	0.014
Yes	963 (12.9)	77 (9.9)	
Transplant center, *n* (%)			
No	5045 (67.6)	572 (73.2)	0.006
Yes	2081 (27.9)	183 (23.4)	
Missing	338 (4.5)	27 (3.5)	
Patient lives in metro vs. non‐metro areas, census tract level			
Metro	6285 (84.2)	677 (86.6)	0.002
Non‐metro	1179 (15.8)	104 (13.3)	
Mean number of ERCP and PTBDs during study period by hospital	164 ± 167.2	145.2 ± 138.7	
45‐min patient accessibility			
Low accessibility: 0.0–2.29	1741 (24.6)	218 (29.3)	0.03
Moderate accessibility: 2.32–3.34	1802 (25.4)	175 (23.6)	
Moderately high accessibility: 3.35–4.13	1762 (24.9)	183 (24.6)	
High accessibility: 4.14–12.62	1779 (25.1)	167 (22.5)	
30‐min patient accessibility			
Low accessibility: 0.0–1.36	1696 (24.9)	202 (27.8)	0.03
Moderate accessibility: 1.38–1.84	1715 (25.1)	176 (24.2)	
Moderately high accessibility: 1.85–2.39	1731 (25.4)	200 (27.6)	
High accessibility: 2.41–7.82	1683 (24.7)	148 (20.4)	
People living in poverty, census tract level (%)			
<5%	973 (13.0)	78 (10.0)	0.03
5%–15%	3596 (48.2)	377 (48.2)	
>15%	2895 (38.8)	327 (41.8)	
Percent of persons with at least 4 years of college, census tract level			
<15%	1553 (20.8)	193 (24.7)	0.004
15%–30%	2728 (36.6)	300 (38.4)	
>30%	3183 (42.6)	289 (37.0)	

Abbreviations: AJCC, American Joint Commission on Cancer; ERCP, endoscopic retrograde cholangiopancreatography; PTBD, percutaneous transhepatic biliary drainage.

### 3.2 Characteristics of study hospitals

There were 808 hospitals in which 8246 patients diagnosed with pancreatic cancer in SEER regions from 2005 to 2013 received a procedure. Among those, 295 hospitals (36.5%) provided both ERCP and PTBD, 492 (63.4%) hospitals provided only ERCP (60.9%), and 21 hospitals (2.5%) provided only PTBD. Hospitals were more likely to only perform one procedure type only (i.e., either ERCP or PTBD) during the study period if they were in a rural location (71.9% vs. urban 44.0%, *p* < 0.001), if they had 300 beds or less (53.9% vs. >300%) beds 35.8%, *p* < 0.001and if they did not have an affiliation with a teaching hospital (54.5% vs. 38.8%, *p* < 0.001).

### 3.3 Association between ERCP access and treatment modality

**FIGURE 1:**
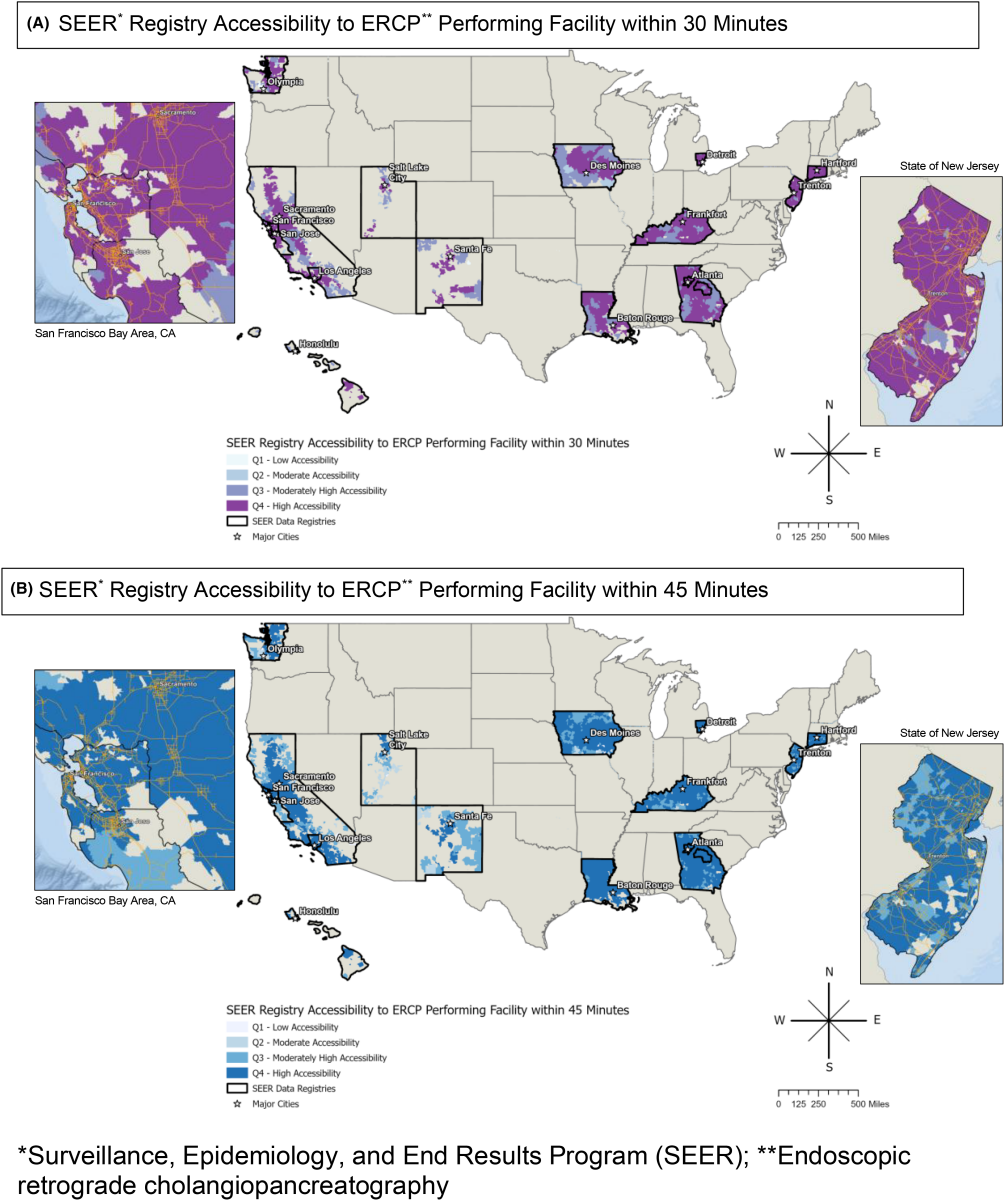
SEER registry accessibility to ERCP‐performing facility within 30 and 45 min. ERCP, endoscopic retrograde cholangiopancreatography; SEER, Surveillance, Epidemiology, and End Results Program.

The majority of our cohort (96.6%) traveled less than 60 min to reach an ERCP‐performing hospital. Accessibility to ERCP‐performing hospitals within both a 30‐ and 45‐min drive varied across and within SEER regions (Figure 1). For example, there is high accessibility to ERCP‐performing hospitals in the Detroit region within both 30 and 45 min; however, in Utah, high accessibility within both 30 and 45 min is limited to areas near Salt Lake City and accessibility is low in more remote areas (Figure 1).

Patients with high accessibility within a 30‐min drive of an ERCP‐performing hospital were significantly more likely to receive an ERCP (Model 1: aOR: 1.50, 95% confidence interval [CI]: 1.15–1.97; Table 2). After adjusting for regional availability of ERCP‐performing hospitals, Black patients (aOR: 0.60, 95% CI: 0.45–0.80), patients with stage IV disease (aOR: 0.57, 95% CI: 0.40–0.80), and patients with GOO (aOR: 0.33, 95% CI: 0.19–0.57) were less likely to receive ERCP (Table 2). There was no significant difference in receipt of ERCP versus PTBD by NCI‐Designated Cancer Center or hospitals organ transplant status (Table 2). Our primary findings were robust to the inclusion of comorbidity as a covariate in a smaller cohort of patients; however, in that model, patients living in metropolitan areas were less likely to receive ERCP (aOR: 0.71, 95% CI: 0.52–0.98; Table 2).

**TABLE 2:** Likelihood of the receipt of ERCP, as compared to PTBD, among patients with pancreatic cancer, multilevel model with 30‐min patient accessibility.

Variable	Model 1^a^ (*n* = 7220)	Model 2 (*n* = 5437)
	OR (95% CI)	OR (95% CI)
Gender		
Male	Ref	Ref
Female	1.08 (0.91–1.28)	1.13 (0.92–1.38)
Age at diagnosis	1.00 (0.99–1.01)	0.99 (0.98–1.01)
Race, *n* (%)		
White	Ref	Ref
Black	0.60 (0.45–0.80)	0.58 (0.42–0.80)
Asian or Pacific Islander	0.98 (0.66–1.45)	1.06 (0.65–1.72)
Other/Unknown/American Indian	0.82 (0.28–2.42)	1.36 (0.34–5.46)
Ethnicity		
Non‐Hispanic	Ref	Ref
Hispanic	0.86 (0.62–1.19)	0.77 (0.53–1.13)
People living in poverty, census tract level (%)		
<5%	Ref	Ref
5%–15%	0.87 (0.64–1.19)	1.07 (0.75–1.52)
>15%	0.90 (0.63–1.29)	0.98 (0.65–1.47)
Percent of persons with at least 4 years of college, census tract level		
<15%	Ref	Ref
15%–30%	1.03 (0.79–1.34)	1.00 (0.74–1.35)
>30%	1.17 (0.86–1.59)	1.19 (0.84–1.70)
AJCC staging, *n* (%)		
Stage 1	Ref	Ref
Stage 2	0.75 (0.53–1.05)	0.70 (0.47–1.04)
Stage 3	0.78 (0.50–1.22)	0.69 (0.42–1.14)
Stage 4	0.57 (0.40–0.80)	0.56 (0.37–0.83)
Unknown	0.66 (0.46–0.96)	0.65 (0.43–0.98)
Procedure year (continuous)		
Per 1 year	1.04 (1.00–1.07)	1.04 (1.00–1.08)
Patients living in metro vs. non‐metro area, census tract level		
Non‐metro	Ref	Ref
Metro	0.75 (0.56–1.01)	0.66 (0.47–0.92)
Charlson comorbidity Index, *n* (%)		
0	–	Ref
1	–	0.94 (0.73–1.21)
2	–	0.96 (0.75–1.23)
Biliary obstruction	1.31 (1.05–1.63)	1.33 (1.03–1.71)
Gastric outlet obstruction	0.33 (0.19–0.57)	0.30 (0.16–0.55)
National Cancer Institute‐Designated Cancer Center	1.08 (0.77–1.51)	1.28 (0.85–1.91)
Organ transplant center	1.13 (0.84–1.54)	1.22 (0.85–1.75)
ERCP + PTBD per hospital (continuous)	1.00 (0.99–1.00)	1.00 (0.99–1.00)
Patient ERCP accessibility 30 min (×10,000)		
Low accessibility: 0.0–1.36	Ref	Ref
Moderate accessibility: 1.38–1.84	1.30 (1.00–1.69)	1.27 (0.94–1.70)
Moderately high accessibility: 1.85–2.39	1.12 (0.87–1.45)	1.13 (0.84–1.51)
High accessibility: 2.41–7.82	1.50 (1.15–1.97)	1.50 (1.10–2.06)

Abbreviations: AJCC, American Joint Commission on Cancer; CCI, Charlson Comorbidity Index; ERCP, endoscopic retrograde cholangiopancreatography; PTBD, percutaneous transhepatic biliary drainage.

^a^ Model 1 did not include CCI due to missing data. Model 2 was estimated for the subgroup of patients with available CCI data.

Results were similar when using a 45‐min drive compared to a 30‐min drive. Patients with high accessibility were more likely to receive ERCP (Model 1: aOR: 1.32, 95% CI: 1.02–1.71; Table 3). Black patients (aOR: 0.59, 95% CI: 0.44–0.80), patients with Stage IV disease (aOR: 0.57, 95% CI: 0.40–0.79) and those with GOO (aOR: 0.33, 95% CI: 0.19–0.57) were less likely to receive ERCP than PTBD. Controlling for comorbidities resulted in the same findings (Table 3, Model 2).

**TABLE 3:** Likelihood of the receipt of ERCP, as compared to PTBD, among patients with pancreatic cancer, multilevel model with 45‐min patient accessibility.

Variable	Model 1^a^ (*n* = 7479)	Model 2 (*n* = 5645)
	OR (99% CI)	OR (95% CI)
Gender		
Male	Ref	–
Female	1.07 (0.90–1.27)	1.13 (0.92–1.38)
Age at diagnosis	1.00 (0.99–1.01)	0.99 (0.98–1.01)
Race, *n* (%)		
White	Ref	Ref
Black	0.59 (0.44–0.80)	0.56 (0.40–0.78)
Asian or Pacific Islander	0.92 (0.62–1.38)	1.01 (0.62–1.65)
Other/Unknown/American Indian	0.86 (0.29–2.55)	1.31 (0.33–5.24)
Ethnicity		
Non‐Hispanic	Ref	Ref
Hispanic	0.82 (0.60–1.14)	0.72 (0.49–1.05)
AJCC staging, *n* (%)		
Stage 1	Ref	Ref
Stage 2	0.76 (0.54–1.06)	0.69 (0.47–1.03)
Stage 3	0.83 (0.54–1.29)	0.71 (0.43–1.18)
Stage 4	0.57 (0.40–0.79)	0.54 (0.36–0.80)
Unknown	0.67 (0.47–0.97)	0.64 (0.42–0.96)
People living in poverty, census tract level (%)		
<5%	Ref	Ref
5%–15%	0.87 (0.64–1.19)	1.04 (0.73–1.49)
>15%	0.90 (0.63–1.30)	0.94 (0.63–1.43)
Percent of persons with at least 4 years of college, census tract level		
<15%	Ref	Ref
15%–30%	1.04 (0.80–1.34)	1.00 (0.74–1.35)
>30%	1.21 (0.89–1.63)	1.20 (0.85–1.71)
Procedure year		
Per 1 year	1.03 (0.99–1.07)	1.03 (0.99–1.08)
Patient lives in metro vs. non‐metro areas, census tract level		
Non‐metro	Ref	Ref
Metro	0.81 (0.61–1.07)	0.75 (0.54–1.03)
Charlson Comorbidity Index, *n* (%)		
0	–	Ref
1	–	0.92 (0.71–1.18)
2	–	0.97 (0.76–1.24)
Obstruction	1.32 (1.06–1.65)	1.35 (1.05–1.75)
Gastric outlet obstruction	0.33 (0.19–0.57)	0.30 (0.16–0.55)
National Cancer Institute Cancer Center	1.10 (0.79–1.54)	1.29 (0.86–1.93)
Transplant center	1.09 (0.80–1.48)	1.17 (0.82–1.67)
ERCP + PTBD per hospital	1.00 (0.99–1.00)	1.00 (0.99–1.00)
Patient accessibility 45 min (×10,000)		
Low accessibility: 0.0–2.29	Ref	Ref
Moderate accessibility: 2.32–3.34	1.24 (0.96–1.61)	1.01 (0.75–1.36)
Moderately high accessibility: 3.35–4.13	1.16 (0.90–1.50)	1.12 (0.84–1.51)
High accessibility: 4.14–12.62	1.32 (1.02–1.71)	1.36 (1.01–1.84)

Abbreviations: AJCC, American Joint Commission on Cancer; CCI, Charlson Comorbidity Index; ERCP, endoscopic retrograde cholangiopancreatography; PTBD, percutaneous transhepatic biliary drainage.

^a^ Model 1 did not include CCI due to missing data. Model 2 was estimated for the subgroup of patients with available CCI data.

Models of patients receiving ERCP with high accessibility within a 60‐min drive had similar findings as the 30‐ and 45‐min models. After adjusting for regional availability of ERCP, Black patients, those with Stage IV disease, and with GOO were less likely to receive ERCP (Table S2). While patients with high accessibility within a 60‐min drive to an ERCP‐performing facility had a greater likelihood of receiving an ERCP, this did not reach statistical significance (aOR: 1.27, 95% CI: 0.98–1.65) (Table S2).

#### 3.3.1 Sensitivity analysis

**FIGURE 2:**
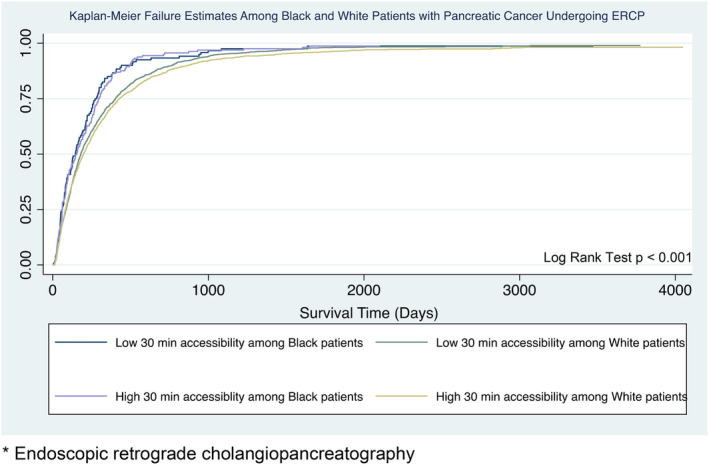
Kaplan–Meier failure estimates comparing Black and White patients undergoing ERCP with both high and low 30‐min accessibility. ERCP, endoscopic retrograde cholangiopancreatography.

Undergoing ERCP was associated with a better overall survival as compared to PTBD (adjusted Hazard Ratio [aHR] 0.72, 95% CI 0.66–0.79) and Black patients had a worse survival than Whites (aHR 1.18, 95% CI 1.08–1.28) in the Cox survival analysis (Table S3). Patient accessibility within 30 min was not associated with survival in our multivariable model. Finally, we compared survival among White and Black patients who underwent ERCP with both low and high 30‐min accessibility to their nearest hospital and found that Black patients had worse overall survival than their White counterparts. (*p* < 0.001, Figure 2).

## 4 DISCUSSION

In our population‐based study, we found that patients with high accessibility to an ERCP‐performing hospital within a 30‐ to 45‐min drive had a greater likelihood of receiving the preferred ERCP treatment as their initial biliary drainage procedure. Similar to prior findings, Black patients were less likely to receive ERCP than PTBD, as were patients with stage IV disease and GOO. Sensitivity analyses including only those patients with a head of pancreas mass, as they may be more likely to require treatment for biliary obstruction during their disease course, produced similar findings.

To our knowledge, this is the first study to characterize geographic access to ERCP‐performing hospitals. It is well‐established that a common barrier to oncological care is patient access to hospitals or physicians providing these specialized services.^20^, ^26^ Geographic access to oncological care is often related in terms of miles or minutes patients travel to hospitals providing specialized care. Studies have found that patients who receive pancreaticoduodenectomy travel a median additional 16.6 miles to reach a high‐volume hospital^22^ and that patients who travel longer distances to reach high‐volume hospitals have improved survival outcomes.^27^ Over 50% of patients with pancreatic cancer with a head mass will require treatment of obstructive jaundice during their disease course and ERCP is a cornerstone of their treatment plan.^28^ Despite this, there have been no studies to date characterizing the geographic distribution of ERCP‐performing hospitals and how geographic access to ERCP is associated with type of biliary procedure received among patients.

We found that patients with the highest access to ERCP‐performing hospitals within a 30‐ to 45‐min drive were more likely to receive ERCP. Although there are no standardized times to evaluate access to hospitals performing specialty procedures, we chose these time frames based on surgical literature that over 75% of patients were traveling less than 1 hour to receive surgery.^26^, ^29^, ^30^ Furthermore, we believe that these findings can have important implications for the distribution of ERCP‐performing hospitals throughout the US. ERCP is a high‐risk procedure and studies have found decreased adverse events among high‐volume endoscopists.^31^, ^32^, ^33^ Because of this thought, leaders in endoscopy have advocated for centralization of ERCP services to high‐volume endoscopists.^31^, ^32^, ^34^ Our study is the first step in understanding the impact of regional centralization of ERCP services in the United States; more studies are needed to understand how regionalization may impact geographic access among noncancer patients.

Furthermore, we found that racial disparities in receipt of ERCP versus PTBD persist even if the patients have the same access to an ERCP‐performing hospital. In the United States, Black patients have both a higher incidence of and a higher mortality from pancreatic cancer.^35^, ^36^, ^37^ Differences in the incidence and mortality of pancreatic cancer are multifactorial and include both modifiable and non‐modifiable risk factors such as alcohol use, cigarette smoking, and genetics.^38^, ^39^, ^40^, ^41^ Prior studies by our group had found that Black patients with pancreatic cancer were less likely to receive ERCP and differential access to ERCP had a negative impact on survival.^15^, ^19^ However, it was unknown whether geographic access to ERCP explained the racial disparities we observed in our prior studies. The results from this study suggest that geographic access to ERCP‐performing hospitals does not explain Black disparities in the receipt of ERCP among older patients with pancreatic cancer. Further work is needed to identify and modify the factors resulting in this racial disparity.

Our study also found that patients with stage IV disease were more likely to receive PTBD than ERCP. While patients with advanced disease are more likely to present with GOO, only 2.2% of patients with stage IV disease in our cohort were diagnosed with GOO. Almost 50% of patients with pancreatic cancer are diagnosed with distant, or stage IV disease, each year.^42^ Many patients with distant, or stage IV, disease will present with obstructive jaundice either due to a head of pancreas mass or metastatic disease from a pancreatic body/tail lesion. While our study could not account for metastatic liver tumor burden among stage IV disease patients, which may be one reason why some patients with stage IV disease preferentially receive PTBD over ERCP, patients with pancreatic cancer without GOO should have an attempt at ERCP, regardless of the stage of their disease.^43^

Our study does have some limitations. First, insurance claims data do not include test results and there may be residual confounding unaccounted for in the SEER‐Medicare database. We performed sensitivity analyses to evaluate the precision of our findings and they were largely unchanged among our analyses. Second, our measure of availability of ERCP was based on our SEER‐Medicare sample with pancreatic cancer. It is possible that there were ERCP providers in SEER regions that we did not measure because they did not perform any ERCPs on Medicare‐insured patients, or because they did not perform ERCPs on patients diagnosed with pancreas cancer during our study period. However, this likely represents a small number of patients given the average age at the time of pancreatic cancer diagnosis is 70.^42^ Third, there are inherent limitations to the methods used to measure accessibility (2SFCA). The 2SFCA method assumes that all people within a hospital's catchment area use the hospital at the same rate despite differences in population characteristics and does not include any other considerations patients may make when deciding where to receive their care, such as insurance. Further, this method lends itself to ecological fallacy, which assumes that what is true for a population is true for the individuals of that population. However, the 2SFCA is one of the most commonly used methods for measuring spatial accessibility.^44^, ^45^ In addition, while our data are from 2005 to 2013, which may make it outdated to the current 2023 ERCP availability, recently published data using MarketScan from 2002 to 2019 found that the rates of ERCP have remained stable from 2002 to 2019.^46^ Similarly, our data show that the number of ERCPs and ERCP‐performing hospitals have remained stable throughout the study period. Finally, our supply‐to‐demand ratio was calculated using the average ERCP volume for ERCP‐performing hospitals across all years during which they performed ERCPs. While this methodology is consistent with prior published literature,^47^ and our study found limited changes in ERCP volumes over our study period, it does not account for temporal changes in availability of ERCP‐performing hospitals and utilization over time, which may play a role in geographic accessibility among patients.

## 5 CONCLUSION

In summary, patients with pancreatic cancer with the highest accessibility to an ERCP‐performing hospital within a 30‐ to 45‐min drive were most likely to receive this optimal treatment for biliary drainage. However, Black patients and those with stage IV disease were more likely to receive PTBD, which is associated with an increased risk of adverse events and decreased survival as compared to ERCP.^9^, ^15^ While our study is the first to evaluate patient access to ERCP services in the United States, more studies are needed to understand both the geographic distribution and access to ERCP for all benign and malignant diseases in the United States and how temporal changes in availability among ERCP‐performing hospitals may influence measures of geographic accessibility.

## AUTHOR CONTRIBUTIONS

**Anna Tavakkoli:** Conceptualization (lead); formal analysis (equal); funding acquisition (lead); writing – original draft (lead). **Alaina Beauchamp:** Methodology (equal); software (equal); writing – review and editing (equal). **Tanushree Prasad:** Methodology (equal); software (equal); writing – review and editing (equal). **Hong Zhu:** Supervision (equal); writing – review and editing (equal). **Amit G. Singal:** Funding acquisition (supporting); supervision (equal); writing – review and editing (equal). **B. Joseph Elmunzer:** Funding acquisition (supporting); supervision (equal); writing – review and editing (equal). **Nisa M. Kubiliun:** Writing – review and editing (equal). **Richard S. Kwon:** Investigation (equal); writing – review and editing (equal). **Amy E. Hughes:** Formal analysis (supporting); methodology (equal); supervision (equal); writing – review and editing (equal). **Sandi L. Pruitt:** Formal analysis (supporting); supervision (equal); writing – review and editing (equal).

## FUNDING INFORMATION

Anna Tavakkoli is funded by NIH 1K23DK132409‐01A1.

## CONFLICT OF INTEREST STATEMENT

Anna Tavakkoli, Alaina Beauchamp, Tanushree Prasad, Hong Zhu, Nisa Kubiliun, Richard Kwon, and Amy Hughes disclose no conflicts of interest. Unrelated to this work, Sandi Pruitt serves as a consultant for Pfizer and Gilead, and Amit Singal has served as a consultant or on advisory boards for Genentech, AstraZeneca, Eisai, Bayer, Exelixis, Boston Scientific, Fujifilm Medical Sciences, Exact Sciences, Roche, Glycotest, Freenome, and GRAIL.

## Supporting information


Figure S1:

Table S1:
Table S2:
Table S3:

## Data Availability

SEER‐Medicare data are available with an approved data use agreement through the National Cancer Institute (https://healthcaredelivery.cancer.gov/seermedicare/). STATA code used to identify cohort, hospitals, and other characteristics are available upon request to the corresponding author.
