# Supplementary figures and images for: Accessibility to ERCP‐performing hospitals among patients with pancreatic cancer living in SEER regions
Source: Cancer Med. 2024 Feb 24;13(3):e7020. doi: 10.1002/cam4.7020 (PMC10891451; doi:10.1002/cam4.7020)

**Supplemental Figure 1: Flowchart of patient cohort**

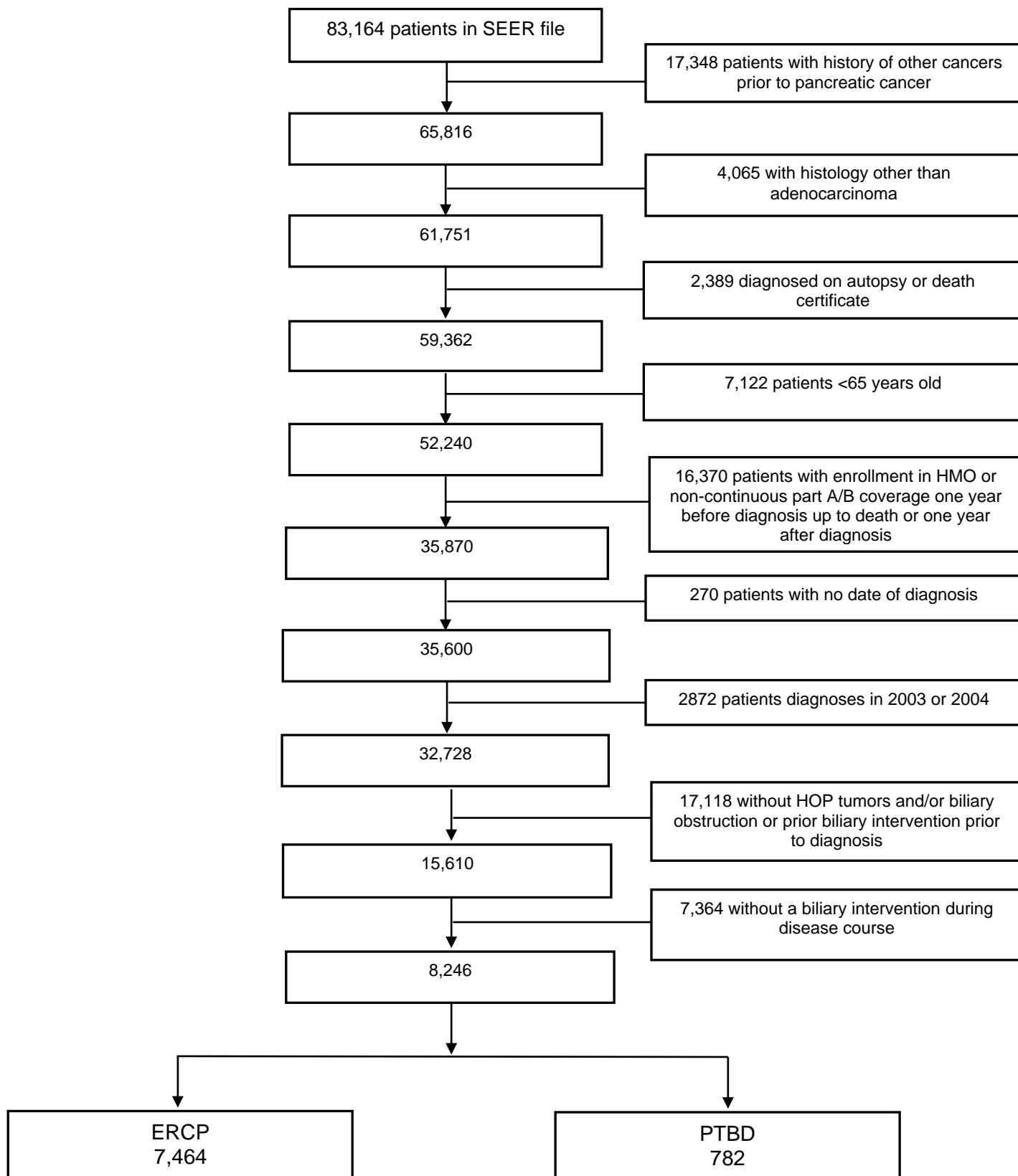

Supplement: Supplementary file 1 — Figure S1: [file CAM4-13-e7020-s002.pdf]
