# Supplementary material for: Accessibility to ERCP‐performing hospitals among patients with pancreatic cancer living in SEER regions
Source: Cancer Med. 2024 Feb 24;13(3):e7020. doi: 10.1002/cam4.7020 (PMC10891451; doi:10.1002/cam4.7020)
Supplement: Supplementary file 2 — Table S1: Table S2: Table S3: [file CAM4-13-e7020-s001.docx]

**Supplemental Table**

Supplemental Table 1: Medicare Billing Codes

| **Variables** | **Codes** |
| --- | --- |
| Pancreatic Adenocarcinoma  (ICD-0-3 codes for histology of pancreatic cancer) | 8000-5, 8010-15, 8020-22, 8050-55, 8140, 8211, 8230, 8260-63, 8440, 8450-53, 8470-71, 8480-81, 8500, 8503, 8521, 8570 |
| ERCP^*^  (HCPCS & ICD-9 procedure codes) | 43260-65, 43271-72, 74328, 74329, 74330, 43267-69, 43274, 43276, 5110, 5111, 5184- 87, 5199, 5213, 5293 |
| PTBD** (HCPCS & ICD-9 procedure codes) | 47505, 47510, 47511, 47525, 75980, 74320, 47533, 47534, 47535, 47536, 47538, 47539, 47540, 8751, 5198 |
| Biliary Obstruction (ICD-9) | 5768, 7824, 5761, 5762, 7948, 6989 |

* Endoscopic retrograde cholangiopancreatography; ** percutaneous transhepatic biliary drainage

Supplemental Table 2: Likelihood of Receipt of ERCP, as compared to PTBD, among Patients with Pancreatic Cancer, multi-level logistic model with 60 minute patient accessibility

| Variable | **Model 1^++^ (n= 7611)** | **Model 2 (n= 5756)** |
| --- | --- | --- |
|  | **OR (99% CI)** | **OR (95% CI)** |
| Gender |  |  |
| Male | Ref |  |
| Female | 1.08 (0.91-1.28) | 1.13 (0.92-1.38) |
| Age at Diagnosis | 1.00 (0.99-1.01) | 0.99 (0.98-1.01) |
| Race, n (%) |  |  |
| White people | Ref | Ref |
| Black people | **0.59 (0.44-0.79)** | **0.56 (0.40-0.78)** |
| Asian or Pacific Islander people | 0.89 (0.60-1.32) | 1.00 (0.60-1.62) |
| Other/Unknown/American Indian | 0.66 (0.27-1.64) | 0.76 (0.27-2.16) |
| Ethnicity |  |  |
| Non-Hispanic | Ref | Ref |
| Hispanic | 0.85 (0.62-1.18) | 0.75 (0.52-1.10) |
| AJCC^*^ Staging, n (%) |  |  |
| Stage 1 | Ref | Ref |
| Stage 2 | 0.76 (0.54-1.06) | 0.70 (0.48-1.04) |
| Stage 3 | 0.83 (0.54-1.29) | 0.68 (0.41-1.11) |
| Stage 4 | **0.57 (0.40-0.79)** | **0.54 (0.37-0.81)** |
| Unknown | **0.67 (0.47-0.97)** | **0.65 (0.43-0.98)** |
| Percent of Persons Living in Poverty, Census tract level |  |  |
| <5% | Ref | Ref |
| 5-15% | 0.88 (0.65-1.20) | 1.07 (0.75-1.52) |
| >15% | 0.94 (0.65-1.33) | 0.99 (0.66-1.49) |
| Percent of Persons with at least 4 years of college, Census tract level |  |  |
| <15% | Ref | Ref |
| 15-30% | 1.03 (0.79-1.33) | 1.00 (0.74-1.34) |
| >30% | 1.21 (0.90-1.64) | 1.21 (0.85-1.71) |
| Procedure Year |  |  |
| Per 1 year | 1.03 (0.99-1.07) | 1.03 (0.99-1.07) |
| Patient lives in Metro vs. Non-Metro census tract |  |  |
| Non-Metro | Ref | Ref |
| Metro | 0.81 (0.61-1.07) | 0.74 (0.54-1.01) |
| Charlson Comorbidity Score, n (%) |  |  |
| 0 | - | Ref |
| 1 | - | 0.91 (0.71-1.16) |
| 2 | - | 0.97 (0.76-1.24) |
| Obstruction | **1.32 (1.06-1.65)** | **1.33 (1.03-1.72)** |
| Gastric Outlet Obstruction | **0.33 (0.19-0.57)** | **0.26 (0.15-0.48)** |
| National Cancer Institute (NCI) Cancer Center | 1.10 (0.79-1.07) | 1.28 (0.85-1.91) |
| Transplant Center | 1.10 (0.81-1.49) | 1.20 (0.84-1.72) |
| ERCP + PTBD per hospital | 1.00 (0.99-1.00) | 1.00 (0.99-1.00) |
| Patient ERCP accessibility 60 min (x 10000) |  |  |
| Low Accessibility : 0.0-2.29 | Ref | Ref |
| Moderate Accessibility: 2.32-3.34 | 1.31 (1.02-1.69) | 1.20 (0.89-1.61) |
| Moderately High Accessibility: 3.35-4.13 | 1.07 (0.84-1.39) | 1.10 (0.82-1.47) |
| High Accessibility: 4.14-12.62 | 1.27 (0.98-1.65) | 1.27 (0.94-1.71) |

*American Joint Commission on Cancer; ^++^Model 1 did not include CCI due to missing data. Model 2 was estimated for the subgroup of patients with available CCI data.

Supplemental Table 3: Factors associated with overall survival among patients with pancreatic cancer, multi-level adjusted Cox model with 30 minute patient accessibility

| Variable | Adjusted Hazard Rate (aHR), 99% CI |
| --- | --- |
| Procedure |  |
| PTBD | Ref |
| ERCP | 0.72 (0.66-0.79) |
| Race |  |
| White people | Ref |
| Black people | 1.18 (1.08-1.28) |
| Gender |  |
| Male | Ref |
| Female | 0.99 (0.93-1.04) |
| AJCC Staging |  |
| Stage 1 | Ref |
| Stage 2 | 0.91 (0.83-1.01) |
| Stage 3 | 1.25 (1.10-1.42) |
| Stage 4 | 2.27 (2.05-2.51) |
| Unknown | 1.50 (1.35-1.67) |
| Age at Diagnosis | 1.03 (1.03-1.04) |
| Charlson Comorbidity Score, n (%) |  |
| 0 | Ref |
| 1 | 1.15 (1.07-1.22) |
| 2 | 1.38 (1.29-1.48) |
| Patient ERCP accessibility 30 min (x 10000) |  |
| Low Accessibility : 0.0-1.36 | Ref |
| Moderate Accessibility: 1.38-1.84 | 0.97 (0.90-1.04) |
| Moderately High Accessibility: 1.85-2.39 | 1.00 (0.92-1.07) |
| High Accessibility: 2.41-7.82 | 1.00 (0.93-1.08) |
